# Angiossarcoma mimetizando *endoleak* tardio pós-reparo endovascular de aneurisma de aorta infrarrenal: relato de caso

**DOI:** 10.1590/1677-5449.004117

**Published:** 2017

**Authors:** Bruno Lorenção de Almeida, Vinicius Pena Caria, Sthefanie Fauve Andrade Cavalcante, Felipe Carvalho Ventin, Eduardo Augusto Moreira Vieira, Eduardo Mulinari Darold, Rodrigo Américo Cunha de Souza, Edmur Carlos Araújo

**Affiliations:** 1 Hospital Santa Helena, Cirurgia Vascular, Brasília, DF, Brasil.; 2 Clínica Eccos, Cirurgia Vascular, Brasília, DF, Brasil.; 3 Hospital Santa Helena, Radiologia, Brasília, DF, Brasil.; 4 Hospital Santa Helena, Cardiologia, Brasília, DF, Brasil.

**Keywords:** angiossarcoma, endoleak, aneurisma

## Abstract

Em todo paciente submetido a reparo endovascular do aneurisma de aorta abdominal (REVA) que se apresente subitamente com quadro de dor abdominal ou sinais de choque, a hipótese de *endoleak* ou vazamento, com expansão do aneurisma e ruptura deve ser aventada. Apresentamos o caso de um paciente em pós-operatório de REVA que apresentou uma neoplasia de duodeno mimetizando um *endoleak*.

## INTRODUÇÃO

O *endoleak* ou vazamento é uma das complicações mais frequentes após o reparo endovascular do aneurisma de aorta abdominal (REVA) infrarrenal, caracterizado pela persistência do fluxo sanguíneo dentro do saco aneurismático, que pode levar à ruptura do aneurisma. Em todo paciente submetido a REVA que se apresente subitamente com quadro de dor abdominal ou sinais de choque, a hipótese de vazamento com expansão do aneurisma e ruptura deve ser aventada. Descrevemos o caso de um paciente em pós-operatório de REVA que apresentou um angiossarcoma mimetizando um *endoleak*.

## DESCRIÇÃO DO CASO

Paciente masculino, 81 anos, com antecedente de hipertensão arterial sistêmica, dislipidemia, diabetes melito e REVA infrarrenal roto contido em 2012 com endoprótese bifurcada, deu entrada no pronto-socorro com queixa de desconforto abdominal intermitente em hipocôndrio direito havia cerca de 3 meses, inapetência e dispneia progressiva aos mínimos esforços. Ao exame físico, apresentou-se desidratado, com mucosas hipocoradas, taquicárdico e taquipneico. Apresentou abdome globoso, flácido e indolor à palpação, com massa não pulsátil e indolor em mesogástrio. Pulsos estavam presentes e simétricos bilateralmente. Exames laboratoriais de entrada mostraram anemia importante e sinais inflamatórios (hemoglobina: 5,2 g/dL, hematócrito: 16,5%, leucócitos: 18.820/mm^3^ sem desvio, plaquetas: 436.000/mm^3^, proteína C-reativa: 155,8 mg/L). O paciente foi estabilizado hemodinamicamente após reposição volêmica e hemotransfusão, e foram solicitadas internação em unidade de terapia intensiva e tomografia de abdome sem contraste. Tinha história prévia de *endoleak* tipo 2 após o REVA e acompanhamento com angiotomografias por 4 anos até a resolução espontânea do vazamento na última tomografia realizada – um total de cinco tomografias, sem nenhuma outra alteração significativa.

**Figura 1:**
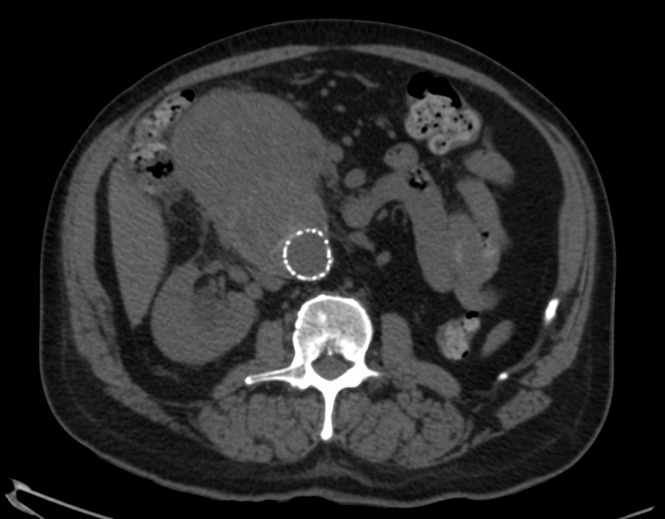
Angiotomografia sem contraste.

**Figura 2:**
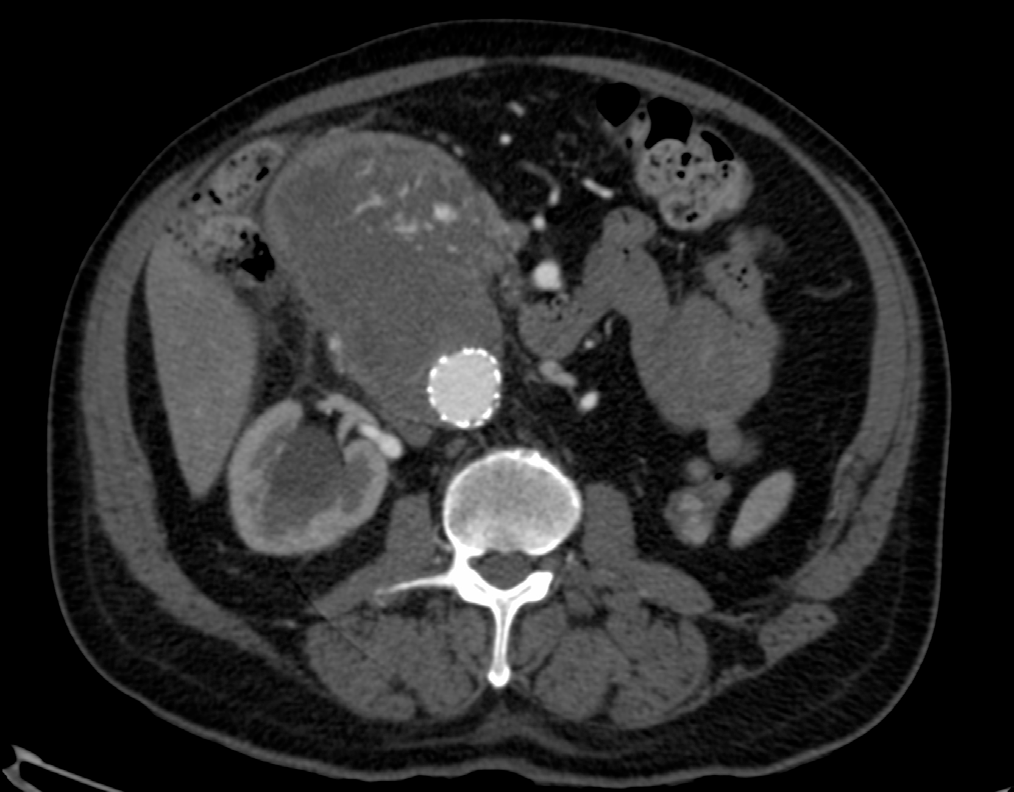
Angiotomografia com contraste evidenciando massa sem plano de clivagem com aorta e com sangramento ativo.

A imagem da tomografia sem contraste evidenciou volumosa massa com densidade de partes moles entre o duodeno e a aorta abdominal, sem plano de clivagem definido (Figura 1). Tendo como principal hipótese um *endoleak* que levou à ruptura contida do aneurisma abdominal e aproveitando o momento de estabilidade clínica do paciente, optamos por realizar a angiotomografia da aorta. Após conversa com o paciente e familiares sobre a importância de exame contrastado e sobre os riscos da nefropatia induzida por contraste, obtivemos seu consentimento. À angiotomografia, notou-se coleção relacionada a segunda e terceira porções do duodeno, adjacente à aorta e ainda sem plano de clivagem definido, medindo cerca 7,3 × 6,2 × 4,9 cm (115 cm^3^), com sangramento ativo para o seu interior (Figura 2). Entretanto, não conseguimos identificar qual seria a origem de um possível vazamento.

**Figura 3:**
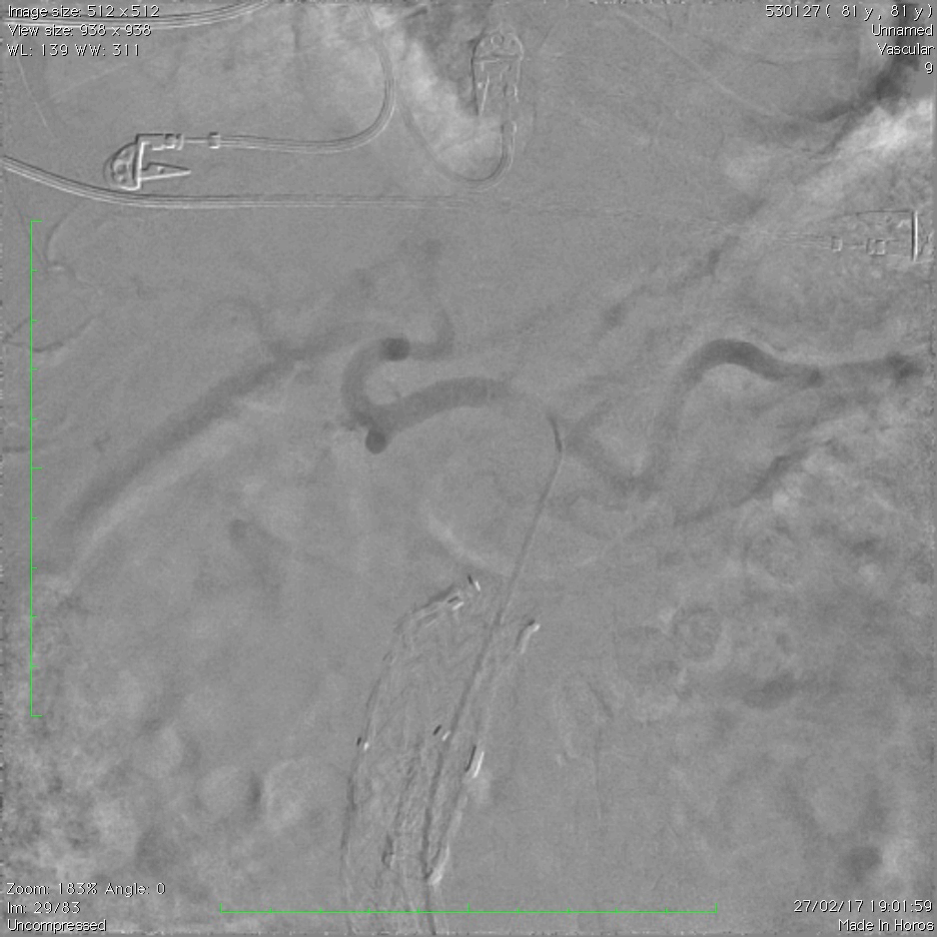
Angiografia seletiva do tronco celíaco.

Optamos por submeter o paciente a angiografia de aorta abdominal e ramos viscerais na tentativa de identificar definitivamente a origem do possível vazamento e embolizar a sua origem. Após cateterização e angiografia seletiva do tronco celíaco e artéria mesentérica superior, não fomos capazes de caracterizar qualquer extravasamento de contraste (Figuras 3 e 4). Injeção de contraste dentro da aorta e no interior da endoprótese também não evidenciou qualquer sinal de *endoleak* (Figura 5).

**Figura 4:**
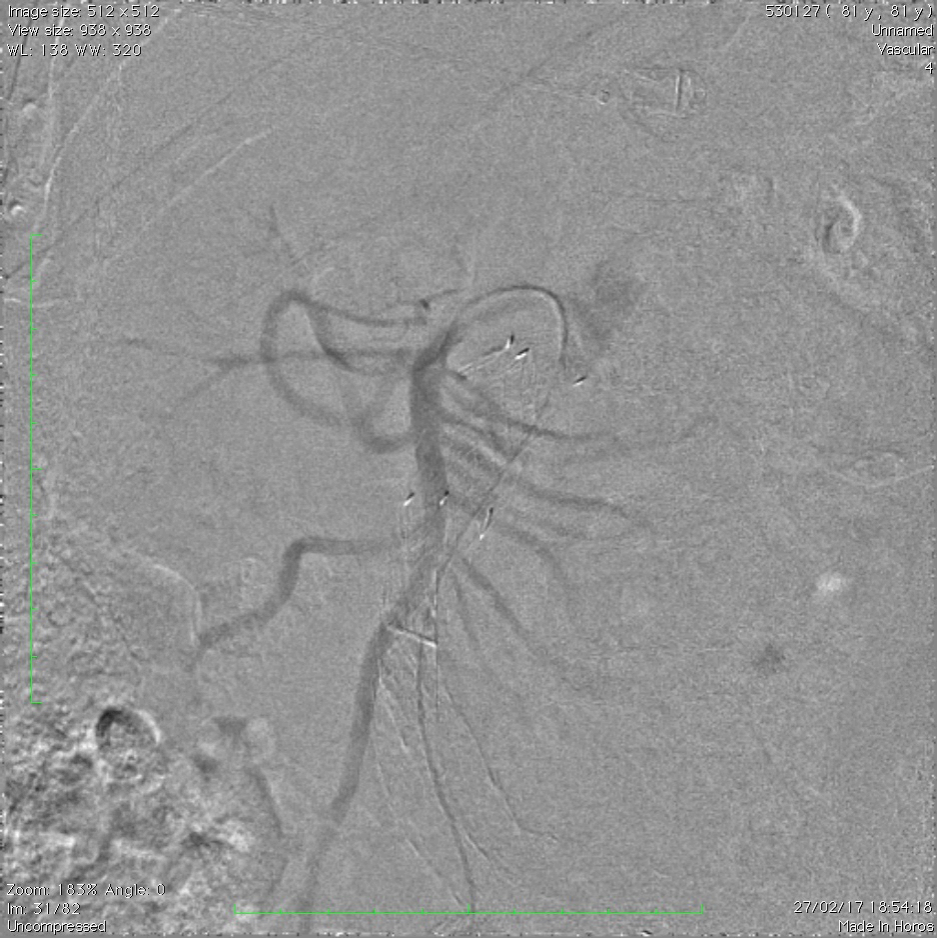
Angiografia seletiva da artéria mesentérica superior.

**Figura 5:**
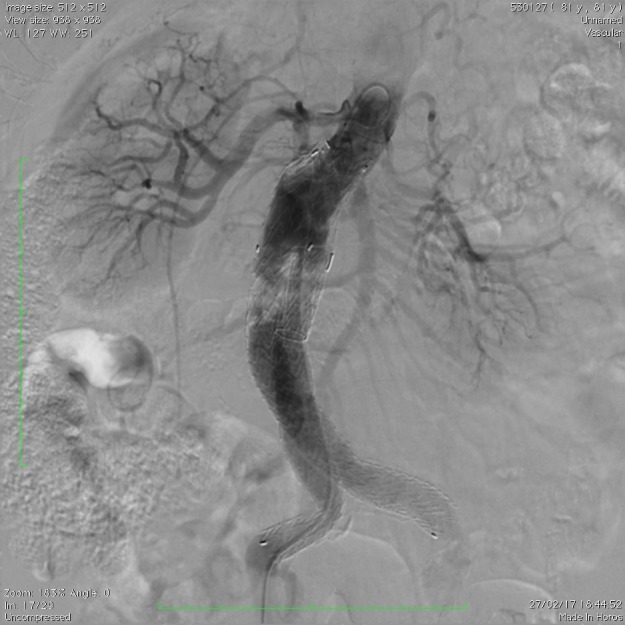
Angiografia da aorta abdominal infrarrenal.

Tendo esgotado as possibilidades diagnósticas invasivas e não sendo capaz de comprovar a existência de vazamento ou ruptura, rediscutimos o caso com a equipe de cirurgia geral, que solicitou endoscopia digestiva alta a fim de investigar a primeira e segunda porções do duodeno. A endoscopia mostrou compressão extrínseca violácea com ponto de sangramento discreto na terceira porção do duodeno, que impediu a passagem do aparelho. No dia seguinte, o paciente fez laparotomia exploradora, que evidenciou tumoração na cabeça de pâncreas e duodeno, sendo submetido a duodenopancreatectomia com linfadenectomia. Em função da idade avançada, comorbidades e porte da cirurgia, o paciente veio a óbito no segundo dia pós-operatório. Exame histopatológico da peça cirúrgica evidenciou angiossarcoma epitelioide de alto grau (neoplasia infiltrativa comprometendo tecido retroperitoneal, pâncreas e parede do duodeno), com margens cirúrgicas profundas comprometidas difusamente e metástase para linfonodo pancreático.

## DISCUSSÃO

O *endoleak* é uma complicação frequente após o REVA, ocorrendo em 15 a 40% dos pacientes submetidos a esse tratamento, frequentemente associado à expansão do aneurisma e necessidade de reintervenção^1^^,^ 2. O *endoleak* tipo 2 é o mais frequente e pode representar até 30% de todos os vazamentos, tendo habitualmente um curso benigno com resolução espontânea durante o acompanhamento. Entretanto, na presença de outros tipos de vazamento, sinais de expansão ou ruptura, o tratamento imediato se impõe^3^^-^ 5. O caso clínico apresentado pelo paciente, associado à imagem angiotomográfica, teve como principal diagnóstico diferencial um *endoleak* associado à ruptura. Entretanto, veio a se revelar como um angiossarcoma mimetizando um *endoleak*.

O angiossarcoma é uma neoplasia maligna rara (1 a 2% de todos os sarcomas) derivada de células endoteliais dos vasos sanguíneos ou linfáticos. Tem predileção por manifestação em pele e tecido celular subcutâneo, seguido por mama, fígado, baço, osso, entre outros. A maioria dos pacientes encontra-se na sexta década de vida, não há predisposição por sexo e, devido ao seu crescimento lento e altamente invasivo, o diagnóstico frequentemente é tardio. Tem como principais fatores patogênicos associados o linfedema crônico; exposição a produtos químicos industriais como arsênico, policloreto de vinila e torotraste; diálise peritoneal por longo período; presença de corpos estranhos e algumas síndromes como neurofibromatose, síndrome de Maffucci, síndrome de Klippel-Trénaunay-Weber, entre outras^6^.

A história clínica apresentada com dor abdominal e anemia à admissão, associada a tratamento endovascular prévio de aneurisma roto, história de acompanhamento clínico de *endoleak* tipo 2 e ausência de plano de clivagem entre a imagem hipervascularizada e o aneurisma à angiotomografia, levou a crer que se tratava de novo vazamento, provocando ruptura contida do aneurisma. Até prova contrária, essa hipótese deveria ser definitivamente excluída pela sua alta morbimortalidade. Entretanto, a angiotomografia seguida de angiografia seletiva falhou em comprovar a existência de qualquer vazamento, o que nos deixou com a hipótese de um processo expansivo tumoral hipervascularizado em topografia de duodeno e cabeça de pâncreas. Sabe-se que a ressonância nuclear magnética com injeção de gadolínio pode ser sensível para diagnóstico de *endoleak* 7 ^,^ 8 – especialmente *endoleak* tipo 2 – e identificação concomitante de processo expansivo tumoral^9^. Entretanto, os artefatos de imagem provocados pela presença da endoprótese metálica no interior da aorta diminuem muito a sensibilidade do método, prejudicando o diagnóstico, e por isso optou-se pela angiotomografia.

Vários artigos mostram a associação do angiossarcoma com grandes vasos, especialmente a aorta, levando a complicações diversas devido à sua alta invasividade: mimetismo de aneurisma de aorta torácica^10^; ruptura de aneurisma de aorta torácica^11^ ou abdominal^12^; hipertensão, anemia e isquemia visceral^13^; mimetismo de infecção de prótese^14^; entre outros. O desenvolvimento de sarcomas associados ao implante de corpos estranhos em humanos e em modelos animais também já foi documentado na literatura^15^. Há relatos de desenvolvimento de angiossarcoma na parede da aorta após correção de aneurisma com implante de endoprótese^16^ e após correção convencional com implante de prótese de dácron^17^. No caso apresentado, entretanto, essa associação não pôde ser apontada dado que o tumor acometeu primariamente o pâncreas e o duodeno, com invasão de um linfonodo pancreático. Não encontramos na literatura caso semelhante ao relatado, no qual o crescimento de um angiossarcoma adjacente à aorta mimetizou um *endoleak*. Fizemos pesquisa na base de dados PubMed utilizando os termos (endoleak[MeSH Terms]) AND angiosarcoma[MeSH Terms], mas não encontramos tal associação.

A exposição repetida à radiação também está documentada na literatura como fator predisponente ao desenvolvimento de angiossarcoma, especialmente em pacientes pós-radioterapia para tratamento do câncer de mama^18^. O paciente em questão realizou o REVA em 2012, em caráter de urgência (aneurisma roto contido), apresentando *endoleak* tipo 2 no pós-operatório. Foram realizadas cinco angiotomografias para acompanhamento do vazamento, sendo que o último exame foi realizado 2 anos antes do evento relatado. Nas imagens, notou-se a resolução do vazamento, sem qualquer sinal de tumoração no duodeno ou pâncreas. Há de se questionar se a exposição repetida do paciente à radiação nas angiotomografias de controle pós-REVA possa ter contribuído para o desenvolvimento e crescimento do angiossarcoma^19^.

O tratamento desses tumores consiste em ressecção cirúrgica com quimioterapia e radioterapia adjuvantes com resultados pouco animadores, visto que a maioria desses tumores é diagnosticada tardiamente. Em estudo publicado por Singla et al., a sobrevida de pacientes submetidos a ressecção tumoral em sua fase inicial foi de 2,33 anos (IC95%: 1,58-14 anos), caindo para 0,92 ano em pacientes submetidos somente a quimioterapia e para 1,0 ano em pacientes tratados somente com radioterapia. Os pacientes com maior sobrevida são aqueles submetidos à cirurgia precocemente, associada à quimioterapia adjuvante e com tumor em estágio menor, sem diferença quanto à sua localização^20^.

## CONCLUSÃO

Apresentamos um caso raro de angiossarcoma epitelioide de alto grau acometendo pâncreas e duodeno, mimetizando um *endoleak* com ruptura aórtica, em paciente previamente submetido a REVA.
