# Supplementary material for: Angiossarcoma mimetizando endoleak tardio pós-reparo endovascular de aneurisma de aorta infrarrenal: relato de caso
Source: J Vasc Bras. 2017 Oct-Dec;16(4):343–7. [Article in Portuguese] doi: 10.1590/1677-5449.004117 (PMC5944315; doi:10.1590/1677-5449.004117)
Supplement: Translation

# Translation: Angiosarcoma mimicking a late endoleak following endovascular aneurysm repair: case report

**Authors:** Bruno Lorenção de Almeida, Vinicius Pena Caria, Sthefanie Fauve Andrade Cavalcante, Felipe Carvalho Ventin, Eduardo Augusto Moreira Vieira, Eduardo Mulinari Darold, Rodrigo Américo Cunha de Souza, Edmur Carlos Araújo

**Affiliations:** 1 Hospital Santa Helena, Cirurgia Vascular, Brasília, DF, Brazil.; 2 Clínica Eccos, Cirurgia Vascular, Brasília, DF, Brazil.; 3 Hospital Santa Helena, Radiologia, Brasília, DF, Brazil.; 4 Hospital Santa Helena, Cardiologia, Brasília, DF, Brazil.

**Keywords:** angiosarcoma, endoleak, anerysm

## Abstract

Whenever a patient who has undergone endovascular repair of an abdominal aortic aneurysm (EVAR) presents with sudden onset abdominal pains or signs of shock, the hypothesis of endoleak with aneurysm expansion and rupture should be considered. We present the case of an EVAR patient in whom a tumor of the duodenum mimicked an endoleak during the postoperative period.

## INTRODUCTION

An endoleak is one of the most common complications observed after endovascular repair of infrarenal abdominal aortic aneurysms (EVAR). It consists of continued blood flow into the aneurysm sac, which can cause the aneurysm to rupture. Whenever a patient who has undergone EVAR presents with sudden onset abdominal pains or signs of shock, the hypothesis of leakage with aneurysm expansion and rupture should be considered. We describe the case of an EVAR patient in whom a neoplasm of the duodenum mimicked an endoleak during the postoperative period.

## CASE DESCRIPTION

An 81-year-old male patient with a history of systemic arterial hypertension, dyslipidemia, diabetes mellitus, and endovascular repair of a ruptured infrarenal abdominal aortic aneurysm, performed in 2012 with a bifurcated endoprosthesis, was admitted to the emergency room complaining of intermittent abdominal discomfort in the right hypochondrium with onset around 3 months previously, lack of appetite, and progressive dyspnea in response to the least effort. On physical examination he was dehydrated, with pale mucosas, tachycardia and tachypnea. His abdomen was distended, flaccid and painless on palpation, with a non-pulsating and painless mass in the mesogastrium. Pulses were present and symmetrical bilaterally. Laboratory tests ordered at admission showed significant anemia and signs of inflammation (hemoglobin: 5.2 g/dL, hematocrit: 16.5%, leukocytes: 18,820/mm^3^ with no shift, platelets: 436,000/mm^3^, C-reactive protein: 155.8 mg/L). Hemodynamic stability was achieved by volume resuscitation and blood transfusion, and he was admitted to the intensive care unit and underwent abdominal tomography without contrast. He had a prior history of type 2 endoleak after the EVAR and had been monitored with angiotomography for 4 years until the leak had resolved spontaneously at the last of five tomographies, which had revealed no other significant findings.

The tomography without contrast images showed a voluminous mass with density of soft tissues between the duodenum and the abdominal aorta, with no clear cleavage plane (Figure 1). Since the principal hypothesis was an endoleak causing a contained rupture of the abdominal aneurysm, and taking advantage of the opportunity provided by the patient’s clinical stability, we decided to conduct an angiotomography of the aorta. After talking to the patient and his relatives about the importance of conducting a scan with contrast and explaining the risks of contrast-induced nephropathy, we obtained consent to proceed. Angiotomography showed accumulation related to the second and third portions of the duodenum, adjacent to the aorta and still with no defined cleavage plane, measuring around 7.3 × 6.2 × 4.9 cm (115 cm^3^), with active bleeding into the interior (Figure 2). However, we were unable to identify the origin of a possible leakage.

**Figure 1:** Angiotomography without contrast.

**Figure 2:** Angiotomography with contrast showing a mass with no plane of cleavage from the aorta and active bleeding.

We decided to conduct angiography of the abdominal aorta and visceral branches in an attempt to definitively identify and embolize the origin of leakage. After selective catheterization and angiography of the celiac trunk and superior mesenteric artery, we were unable to discern any leakage of contrast (Figures 3 and 4). Injection of contrast into the aorta and the interior of the endoprosthesis also failed to reveal any evidence of endoleak whatsoever (Figure 5).

**Figure 3:** Selective angiography of the celiac trunk.

**Figure 4:** Selective angiography of the superior mesenteric artery.

**Figure 5:** Angiography of the infrarenal abdominal aorta.

Since we had exhausted the invasive diagnostic options without being able to confirm the existence of leakage or rupture, we discussed the case with the general surgery team again. They ordered an upper digestive endoscopy in order to investigate the first and second portions of the duodenum. The endoscopy showed purple extrinsic compression with a discrete source of bleeding in the third portion of the duodenum, which impeded progression of the endoscope. The following day, an explorative laparotomy found evidence of a tumor at the head of the pancreas and duodenum and duodenopancreatectomy with lymphadenectomy were performed. The patient died two days after the operation because of advanced age, comorbidities, and the large scale surgery. Histopathological examination of the surgical specimen revealed high grade epithelioid angiosarcoma (an invasive neoplasm compromising retroperitoneal tissues, pancreas, and the wall of the duodenum), deep surgical margins with diffuse compromise and metastasis to the pancreatic lymph node.

## DISCUSSION

Endoleaks are a common complication following EVAR, occurring in 15 to 40% of patients who undergo this treatment and often linked with expansion of the aneurysm and a need for reintervention.^1^^,^ 2 Type 2 endoleaks are the most common and can account for up to 30% of all leaks and habitually have a benign outcome with spontaneous resolution during follow-up. However, when there are also other types of leakage, or signs of expansion or rupture, immediate treatment is obligatory.^3^^-^ 5 The clinical presentation of this patient, which was compounded by the angiotomographic image, suggested a principal differential diagnosis of endoleak associated with aneurysm rupture. However, further investigation revealed that it was actually the result of an angiosarcoma mimicking an endoleak.

Angiosarcoma is a rare malignant neoplasm (1 to 2% of all sarcomas) derived from blood vessel or lymphatic endothelial cells. It has a preference for skin and subcutaneous cellular tissues, followed by the breasts, liver, spleen, and bone, among other possible sites. The majority of patients are in their sixth decade of life, there is no predisposition for either sex, and its slow and highly invasive growth means that diagnosis is often late. The principal pathogenic factors associated with it are chronic lymphedema; exposure to industrial chemicals such as arsenic, polyvinyl chloride, and thorotrast; long-term peritoneal dialysis; presence of foreign bodies; and certain syndromes such as neurofibromatosis, Maffucci syndrome, Klippel-Trénaunay-Weber syndrome, and others.^6^

The clinical history with abdominal pains and anemia at presentation, combined with prior endovascular treatment for a ruptured aneurysm, history of clinical monitoring of a type 2 endoleak, and the absence of a cleavage plane between the hypervascularized image and the aneurysm on angiotomography led to the belief that there had been another leak, provoking a contained rupture of the aneurysm. This hypothesis should be pursued until it can be definitively ruled out because of its high rates of morbidity and mortality. However, angiotomography followed by selective angiography failed to confirm the existence of any type of leakage, which left us with the hypothesis of an expanding hypervascularized tumor in the area of the duodenum and the head of the pancreas. It is known that magnetic resonance imaging with gadolinium injection can be sensitive for diagnosis of endoleak^7^^,^ 8 – especially type 2 endoleak – and concurrent identification of an expanding tumor process.^9^ However, the imaging artifacts provoked by the presence of the metallic endoprosthesis in the interior of the aorta greatly reduced the sensitivity of the method, compromising the diagnosis, and so the decision was taken to conduct angiotomography.

There are several articles describing angiosarcoma involving large vessels, especially the aorta, and causing a range of complications because of its high invasivity: mimicking thoracic aortic aneurysm^10^; rupture of aneurysms of the thoracic aorta^11^ or abdominal aorta^12^; hypertension, anemia and visceral ischemia^13^; and mimicking an infection of a prosthesis^14^; among others. Development of sarcomas has also been documented in the literature in association with implantation of foreign bodies in humans and in animal models.^15^ There are reports of development of angiosarcoma in the aorta wall after aneurysm repair with implantation of an endoprosthesis^16^ and after conventional repair with implantation of a dacron graft.^17^ This association cannot be attributed to the case described here, however, since the tumor primarily involved the pancreas and the duodenum, with invasion of a pancreatic lymph node. We could not find any cases in the literature similar to this one, in which growth of an angiosarcoma adjacent to the aorta mimicked an endoleak. We searched the PubMed database using the terms (endoleak[MeSH Terms]) AND angiosarcoma[MeSH Terms], but did not find any instances of this combination.

Repeated exposure to radiation is also documented in the literature as a factor predisposing to development of angiosarcoma, especially in patients who have had radiotherapy to treat breast cancer.^18^ The patient described here underwent emergency EVAR in 2012 (for a contained ruptured aneurysm) and was found to have a type 2 endoleak during the postoperative period. He underwent five angiotomographies to monitor the leak, the last of which was conducted 2 years before the event described here. Those images showed the leakage with no sign whatsoever of tumors in the duodenum or pancreas. The question must be raised as to whether repeated exposure of the patient to radiation for control angiotomographies after EVAR may have contributed to development and growth of the angiosarcoma.^19^

Treatment for these tumors consists of surgical resection with adjuvant chemotherapy and radio therapy, with fairly dismal results, since the majority of these tumors are diagnosed at late stages. In a study published by Singla et al., mean survival of patients who underwent tumor resection during the initial phase was 2.33 years (95%CI: 1.58-14 years), falling to 0.92 years in patients treated with chemotherapy alone and to 1.0 year in patients treated with radiotherapy only. The highest survival rates are for patients who undergo surgery early, combined with adjuvant chemotherapy and with tumors at lower stages, while tumor site is unrelated to differences in survival.^20^

## CONCLUSIONS

We have presented a rare case of high grade epithelioid angiosarcoma involving the pancreas and duodenum and mimicking an endoleak with aortic rupture, in a patient who had previously undergone EVAR.
